# Patient-Specific Modeling of Stented Coronary Arteries Reconstructed from Optical Coherence Tomography: Towards a Widespread Clinical Use of Fluid Dynamics Analyses

**DOI:** 10.1007/s12265-017-9777-6

**Published:** 2017-12-27

**Authors:** Claudio Chiastra, Susanna Migliori, Francesco Burzotta, Gabriele Dubini, Francesco Migliavacca

**Affiliations:** 1 Laboratory of Biological Structure Mechanics (LaBS), Department of Chemistry, Materials and Chemical Engineering “Giulio Natta”, Politecnico di Milano, Piazza Leonardo da Vinci 32, 20133 Milan, Italy; 2 Institute of Cardiology, Catholic University of the Sacred Heart, Rome, Italy

**Keywords:** Optical coherence tomography, Coronary artery, Stent, Image segmentation, Image processing, Computer simulations, Computational fluid dynamics, In silico clinical trial

## Abstract

The recent widespread application of optical coherence tomography (OCT) in interventional cardiology has improved patient-specific modeling of stented coronary arteries for the investigation of local hemodynamics. In this review, the workflow for the creation of fluid dynamics models of stented coronary arteries from OCT images is presented. The algorithms for lumen contours and stent strut detection from OCT as well as the reconstruction methods of stented geometries are discussed. Furthermore, the state of the art of studies that investigate the hemodynamics of OCT-based stented coronary artery geometries is reported. Although those studies analyzed few patient-specific cases, the application of the current reconstruction methods of stented geometries to large populations is possible. However, the improvement of these methods and the reduction of the time needed for the entire modeling process are crucial for a widespread clinical use of the OCT-based models and future in silico clinical trials.

## Introduction

Over the last years, the use of computer modeling as a tool for new medical device development and pre-operative planning has become more and more important in the biomedical field. Both the Food and Drug Administration (FDA) in the USA and the European Commission have recently started to support this technology, which is expected to dramatically cut the cost of bringing a new device to the market in the near future (from US$ 2.5bn to US$ 250m by 2025) [1]. In fact, it is believed that in silico clinical trials, defined as “the use of individualized computer simulation in the development or regulatory evaluation of a medicinal product, medical device, or medical intervention” [2], could help in reducing, refining, and partially replacing real clinical trials, resulting in a significant reduction of their associated direct and indirect costs [1].

One of the key success factors of an in silico clinical trial is the creation of anatomically accurate and validated patient-specific models. The recent widespread application of optical coherence tomography (OCT) in interventional cardiology has improved patient-specific modeling of stented coronary arteries. OCT is an intravascular catheter-based imaging modality that uses near-infrared light and acquires cross-sectional vessel images by interferometry [3]. The high resolution of OCT images (axial resolution of 12 ± 15 μm, lateral resolution of 20 ± 40 μm, and penetration depth in the vessel wall of 1–2.5 mm [4]) enables the detection of both lumen contours and stent struts. This paves the way for the reconstruction of detailed three-dimensional (3D) geometrical models of coronary arteries that include a high fidelity stent geometry. Such geometries are suitable for analyzing the local hemodynamics in the proximity of the stent struts using computational fluid dynamics (CFD) simulations. In particular, the high level of geometrical detail enables the quantification of the altered wall shear stress (WSS) pattern caused by the stent struts.

Cumulative evidence has highlighted that the altered local hemodynamics is an important factor modulating the pathophysiological mechanisms that can lead to thrombosis or in-stent restenosis [5]. Briefly, regarding thrombosis, in vitro perfusion experiments in stented coronary artery phantoms have quantified the impact of blood flow and high shear rate on clot formation [6, 7]. Regarding restenosis, in vitro experiments have demonstrated a direct action of WSS on endothelial cell morphology, orientation, and function [8, 9]. At a macro-level, animal studies have shown that tissue regrowth in stented arteries is prominent at sites of low and/or oscillatory WSS [9–11]. Although limited by the small number of analyzed cases, some patient studies have found a similar relationship between neointimal regrowth and altered WSS pattern [12]. These evidences explain the recent research interest on the accurate quantification of patient-specific stented artery hemodynamics as a means to predict stent failure induced by thrombosis or in-stent restenosis in the clinical setting.

The present review focuses on the OCT-based patient-specific modeling of stented coronary arteries for the analysis of the local hemodynamics. Particular attention is dedicated to aspects such as the validation of the reconstruction methods, the boundary conditions of the CFD models, and the duration of the entire modeling process, which are important to make these computational models appropriate for a widespread and sustainable clinical application and future in silico clinical trials.

**Fig. 1:**
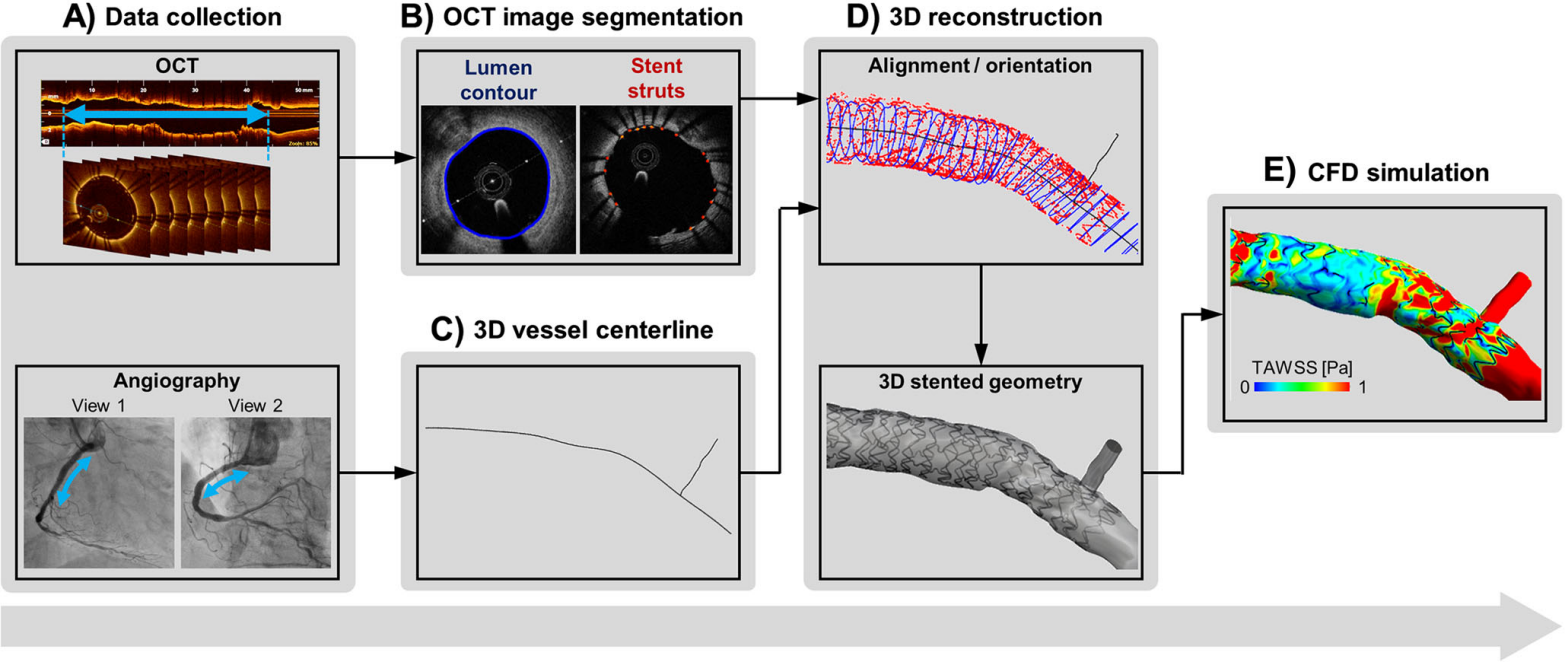
Workflow for the creation of patient-specific stented coronary artery models from OCT images: A) collection of patient’s clinical data, B) detection of lumen contours and stent struts from OCT images using automatic segmentation algorithms, C) extraction of vessel centerline from angiography (or computed tomography), D) 3D reconstruction of stented geometries by combining the detected lumen contours and stent struts with the vessel centerline, E) execution of CFD simulations

As shown in Fig. 1, the main steps for the creation of a patient-specific stented coronary artery model from OCT images are (1) the collection of a patient’s clinical data, including OCT and angiography (or computed tomography—CT); (2) the detection of lumen contours and stent struts from OCT images using automatic segmentation algorithms; (3) the 3D reconstruction of the stented geometry by combining OCT and angiography (or CT); and (4) the execution of the CFD simulation. The following sections describe these steps, focusing in particular on the state of the art of the segmentation methods of OCT images (step 2) and the CFD models (steps 3 and 4). Furthermore, the limitations of the current modeling strategy and the future perspectives are discussed.

## Automatic Segmentation Methods of OCT Images

### State of the Art

Recently, many segmentation algorithms for automatic detection of lumen contours and stent struts from OCT images have been proposed. Table 1 reports a list as comprehensive as possible of the studies published on this topic. While several studies presented a detection algorithm of the lumen contours [13, 15, 18, 19, 22, 26, 30, 31, 35, 36, 39, 40] or the stent struts [20, 21, 23, 24, 27, 29, 32] only, in other works both detection algorithms were developed [14, 16, 17, 25, 28, 33, 34, 37, 38, 41, 42].

**Table 1:** List of published studies on (semi-)automatic lumen and stent strut detection algorithms of coronary artery OCT images

First author, year [reference]	Lumen segmentation	Stent segmentation	Software/programming language	Calculation time (hardware)	Ground truth for validation	Number of OCT pullbacks (frames)	OCT system
Sihan et al., 2009 [13]	Yes	No	Matlab	2–5 s/frame (ND)	Manual segmentation	20 pullbacks from 20 patients (4137 frames)	LightLab Imaging, Inc.
Gurmeric et al., 2009 [14]	Yes	Yes	ND	ND (ND)	Manual segmentation	7 pullbacks from 7 patients (39 frames)	M2 (LightLab Imaging, Inc.)
Wang Z. et al., 2010 [15]	Yes	No	ND	ND (ND)	Manual segmentation	9 pullbacks from 8 patients (63 frames)	M4 (LightLab Imaging, Inc.)
Unal et al., 2010 [16]	Yes	Yes	ND	ND (ND)	Manual segmentation	7 pullbacks from 7 patients (39 frames)	M2 (LightLab Imaging, Inc.)
Kauffmann et al., 2010 [17]	Yes	Yes	Interface description language	0.8 s/frame (Intel Pentium 4 3.4 GHz, 1 GB RAM)	Manual segmentation	11 pullbacks from 11 patients, 1 pullback from 1 phantom (stented urinary catheter) (frames ND, 3588 struts)	M2 (LightLab Imaging, Inc.)
Tung et al., 2011 [18]	Yes	No	ND	ND (ND)	Manual segmentation	4 pullbacks from 4 patients (ND)	M4 (LightLab Imaging, Inc.)
Wang Z. et al., 2011 [19]	Yes	No	ND	ND (ND)	Manual segmentation	9 pullbacks from 8 patients (63 frames)	M4 (LightLab Imaging, Inc.)
Xu et al., 2011 [20]	No	Yes	ND	ND (ND)	Manual segmentation	9 pullbacks from 9 patients (613 frames)	C7-XR (LightLab Imaging, Inc.)
Bruining et al., 2011 [21]	No	Yes	ND	4.1 s/frame for the post-implant group (ND) 6.3 s/frame for the follow-up group (ND)	Manual segmentation	29 pullbacks (4024 frames)	LightLab Imaging, Inc.
Athanasiou et al., 2012 [22]	Yes	No	ND	ND (ND)	Not performed	1 pullback from 1 patient (ND)	LightLab Imaging, Inc.
Lu et al., 2012 [23]	No	Yes	ND	~ 9 s/frame (ND)	Manual segmentation	6 pullbacks from 6 patients (frames ND)	C7-XR (LightLab Imaging, Inc.)
Tung et al., 2012 [24]	No	Yes	ND	ND (ND)	Manual segmentation	4 pullbacks from 4 patients (frames ND)	C7-XR (LightLab Imaging, Inc.)
Ughi et al., 2012 [25]	Yes	Yes	Matlab, C++	< 1 s/frame (ND)	Manual segmentation	9 pullbacks from 9 patients (108 frames)	C7-XR (LightLab Imaging, Inc.)
Moraes et al., 2013 [26]	Yes	No	Matlab	5.9 ± 3 s/frame (Intel Core 2 Duo 2.53 GHz, 4 GB RAM)	Manual segmentation	5 pullbacks from 2 patients, 2 pigs, 1 rabbit (290 frames)	LightLab Imaging, Inc.
Wang A. et al., 2013 [27]	No	Yes	MeVisLab toolbox, C++	< 1.1 s/frame (2.0 GHz CPU, 4 GB RAM)	Manual segmentation	10 pullbacks from 7 patients (3231 frames, 18,311 struts)	C7-XR (LightLab Imaging, Inc.)
Han et al., 2013 [28]	Yes	Yes	Intel IPP library on CPU, CUDA technology on GPU	< 0.279 s/frame (ND)	Manual segmentation	3 pullbacks from 3 patients (369 frames, 3712 struts)	ND
Wang A. et al., 2014 [29]	No	Yes*	MeVisLab toolbox, C++	ND	Manual segmentation	6 pullbacks from 6 patients (frames ND, 4691 struts)	C7-XR (LightLab Imaging, Inc.)
Celi and Berti, 2014 [30]	Yes	No	Matlab	13 s/frame (Intel Core 2 Quad processors (2.67 GHz, 4 GB RAM)	Manual segmentation	10 pullbacks from 10 patients (2800 frames)	C7-XR (LightLab Imaging, Inc.)
Chatzizisis et al., 2014 [31]	Yes	No	Matlab	< 1 s/frame (ND)	Manual segmentation	20 pullbacks from 20 patients (1682 frames)	C7-XR (LightLab Imaging, Inc.)
Wang Z. et al., 2015 [32]	No	Yes	Matlab, C++	~ 1.5 s/frame (duo-core 3.0 GHz)	Manual segmentation	103 patients from 78 pullbacks (8000 frames)	C7-XR (St. Jude Medical)
Dubuisson et al., 2015 [33]	Yes	Yes	ND	Few minutes per pullback (ND)	Manual segmentation	4 pullbacks from 4 patients (77 frames)	C7-XR (LightLab Imaging, Inc.)
Han et al., 2015 [34]	Yes	Yes	Visual Studio, CUDA 5.0, Qt 5.0, VTK 6.0	< 0.1 s/frame (Intel Xeon E5-2630 2.30 GHz, 2 Nvidia GTX680, 32 GB RAM)	Manual segmentation	5 pullbacks from 5 patients (305 frames)	ND
de Macedo et al., 2016 [35]	Yes	No	Matlab	15 s/frame (Intel i7 3.46 GHz, 32 GB RAM)	Manual segmentation	9 pullbacks from 9 patients (1328 frames)	C7-XR (St. Jude Medical)
Guha Roy et al., 2016 [36]	Yes	No	Matlab	18.82 ± 1.77 s/frame (Intel i3 2.50 GHz, 4 GB RAM)	Manual segmentation	15 pullbacks from 15 ex vivo human arteries, 6 pullbacks from 6 patients (ND)	C7-XR (St. Jude Medical)
Nam et al., 2016 [37]	Yes	Yes	Matlab	~ 0.47 s/frame (Intel Pentium G850 2.9 GHz, 8 GB RAM)	Manual segmentation	20 pullbacks from 18 patients (800 frames)	C7-XR (St. Jude Medical)
O’Brien et al., 2016 [38]	Yes	Yes	ND	ND (ND)	Manual segmentation	4 pullbacks from 4 pigs (62 frames for the lumen, 57 frames for the stent)	C7-XR (St. Jude Medical)
Cao et al., 2017 [39]	Yes	No	ND	ND (ND)	Against method by Ughi et al. [32]	5 pullbacks from 5 patients (880 frames)	C7-XR (St. Jude Medical)
Cheimariotis et al., 2017 [40]	Yes	No	ND	1 s/frame (ND)	Manual segmentation	20 pullbacks from 20 patients (1812 frames)	C7-XR (St. Jude Medical)
Chiastra et al., 2017 [41]	Yes	Yes	Matlab	~ 0.55 s/frame (Intel i7-950 3.07 GHz and 16 GB RAM)	Manual segmentation Micro-CT	14 pullbacks from 8 coronary bifurcation phantoms (160 frames), 4 pullbacks from 4 patients	C7-XR (St. Jude Medical)
Migliori et al., 2017 [42]	Yes	Yes	Matlab	ND (ND)	Manual segmentation	1 pullback from 1 coronary phantom (95 frames for the lumen, 120 frames for the stent)	C7-XR (St. Jude Medical)

*ND* not declared

*Algorithm applied to polymeric bioresorbable scaffolds

#### Lumen Contour Detection

**Fig. 2:**
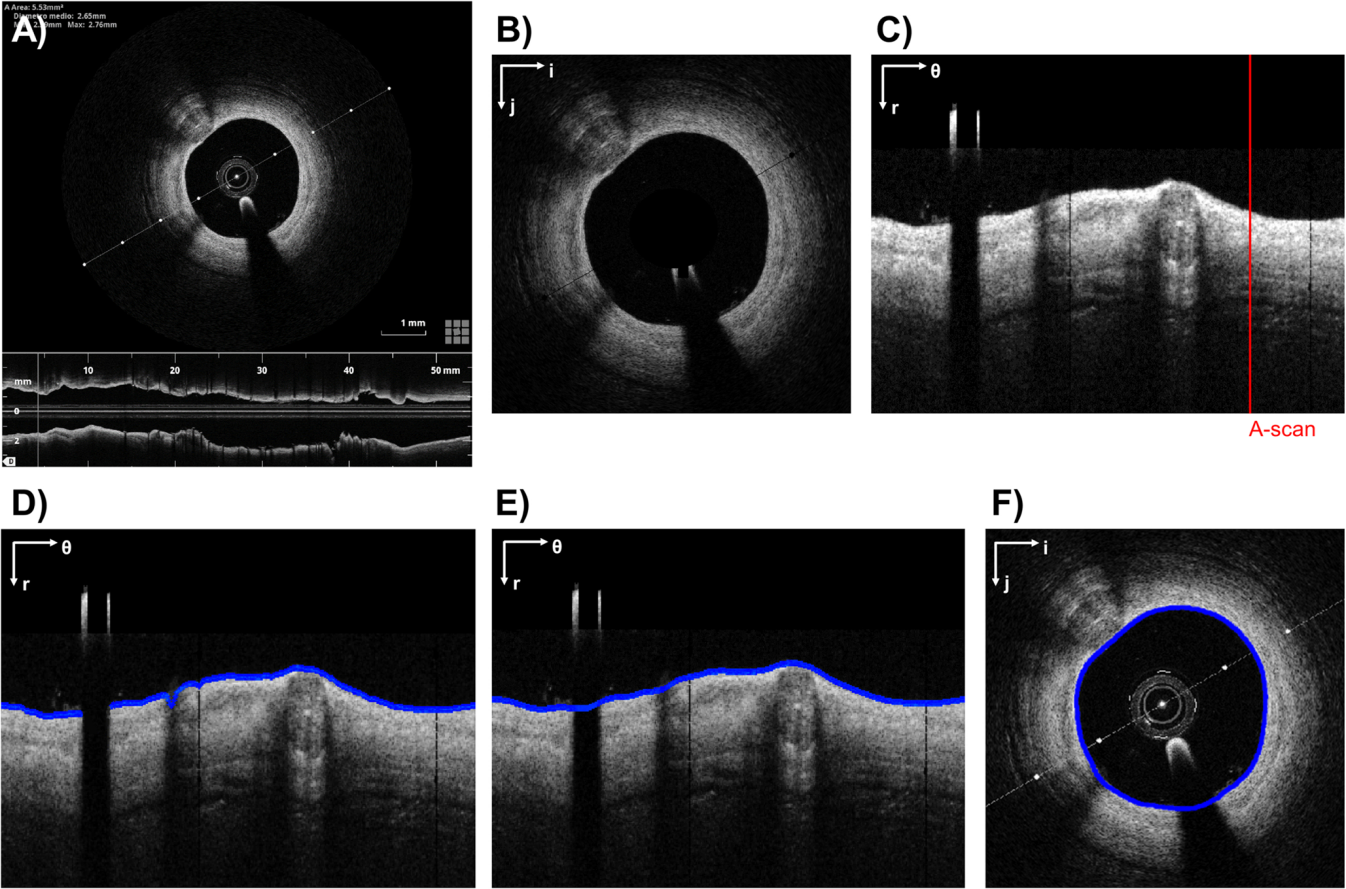
Example of lumen detection algorithm. **a** Original grayscale OCT image. **b** Pre-processed image without OCT catheter. **c** Pre-processed image in polar coordinates. The red line highlights an example of A-scan. **d** Raw lumen contour detection (blue). **e** Lumen contour (blue) detected after gap closing and smoothing. **f** Lumen contour (blue) after conversion to Cartesian coordinates. The polar coordinate system (*r*; *θ*) or the Cartesian coordinate system (*i*; *j*) is indicated on the top left of each image. The example refers to a post-operative OCT image of a patient treated at the Institute of Cardiology, Catholic University of the Sacred Heart (Rome, Italy), with a Xience Prime stent (Abbott Vascular, USA). The image was processed using the algorithm described in [41, 42]

The workflow for the detection of lumen contours usually consists of three steps: (1) pre-processing, (2) contour extraction, and (3) contour refinement. An example of the entire workflow is shown in Fig. 2.

During the pre-processing (Fig. 2a, b), the OCT catheter cross section is removed. Image binarization is then performed by setting a threshold to enhance the desired information in the image. Different thresholds have been proposed in the literature. For instance, in several studies [14, 16, 31, 41, 42], an appropriate percentile of the image intensity histogram was chosen as threshold; in other studies [15, 17, 22, 26, 28, 30, 33–35, 40], Otsu’s thresholding method [43] was applied. A smoothing filter (e.g., median filter) and/or morphological operations are finally used to further enhance and smooth the image by removing speckle noise and filling small gaps within the vessel wall. When raw imaging data are not available, the images processed by the OCT system, which are often compressed, can be used as an alternative. In this case, the previous operations have to be preceded by the cropping of the lower part of each OCT frame, which represents the longitudinal vessel view (Fig. 2a), and the removal of all pixels belonging to OCT visualization tools (e.g., line representing the section plane of the longitudinal section and scale). However, wherever possible, the imaging processing should be always performed on the raw OCT images to guarantee the best segmentation result.

Lumen contour extraction relies on the morphology of the pre-processed image. In general, the OCT image pixels have different intensity levels within the vessel wall thickness, from higher values at the inner face of the vessel wall to lower values at the outer vessel wall layers. In the majority of proposed algorithms [19, 22, 25, 26, 28, 31, 33–35, 37–42], the image is converted into polar coordinates to facilitate the description of local image regions in terms of their radial and tangential characteristics (Fig. 2c) [44]. The strategies proposed for the lumen detection are various. One strategy consisted of analyzing each A-scan (i.e., one-dimensional depth profile, Fig. 2c) by moving from the OCT catheter to the vessel wall (i.e., from top to bottom of the image) to search for the pixel with the maximum intensity and by selecting the pixel previous to that with maximum intensity for the definition of the lumen contour [25]. A similar intensity-based approach was followed by other studies [15, 22, 28, 34, 35, 40]. Wang et al. [19, 32] solved an optimization problem by looking for the contour that maximizes the intensity difference between the sum of gray values outside and inside the lumen. Dubuisson et al. [33] used Dijkstra’s algorithm [45], based on the graph theory, to find the lumen contour as the shortest path from the left to the right of the polar image. Nam et al. [37] extracted the lumen contour by detecting and modifying a temporary lumen contour, which was obtained by connecting inward points at 20% of the A-scans’ maximum intensity. O’Brien et al. [38] first classified the A-scans of each OCT frame as belonging to a lumen using features extracted from the “scale-space signature” of the A-scan signal. This signature was generated by convolving the A-scan signal with a specific mother wavelet. Then, similarly to the Canny edge detector [46], the location of the lumen contour within each A-scan was detected by convolving the A-scan signal with the normalized first derivative of the Gaussian wavelet. Cao et al. [39] implemented a level-set-based method, which iteratively adapts an initial segmentation to the real lumen contour in polar coordinates. Chiastra et al. [41] and Migliori et al. [42] proposed a gradient-based approach that applies a Sobel edge detection filter [47] to the pre-processed image and accepts the first non-zero pixel in each A-scan as a valid point for lumen contour (Fig. 2d). Differently from the previously reported studies, which process the image in the polar domain, other algorithms exclusively work in the Cartesian domain. In particular, a number of studies [14, 16, 17] proposed an active contour-based method in which a Catmull-Rom spline was initialized inside the lumen and propagated towards the lumen contour until it reached the desired boundary; other studies [18, 36] used a graph-cut-based segmentation method to extract the lumen contour; Sihan et al. [13] applied the Canny edge detector [41] to find edges and then a heuristic method to exclude some of them and link others into the lumen contour.

During the last step of the detection algorithm, the segmented lumen contour is interpolated and smoothed to generate a continuous contour (Fig. 2e). In particular, the gap caused by the OCT catheter shadow is filled. Finally, as regards those algorithms that perform contour extraction in the polar domain [19, 22, 25, 26, 28, 31, 33–35, 37–42], the image is converted back to Cartesian coordinates to obtain the final closed lumen contour (Fig. 2f).

#### Stent Strut Detection

**Fig. 3:**
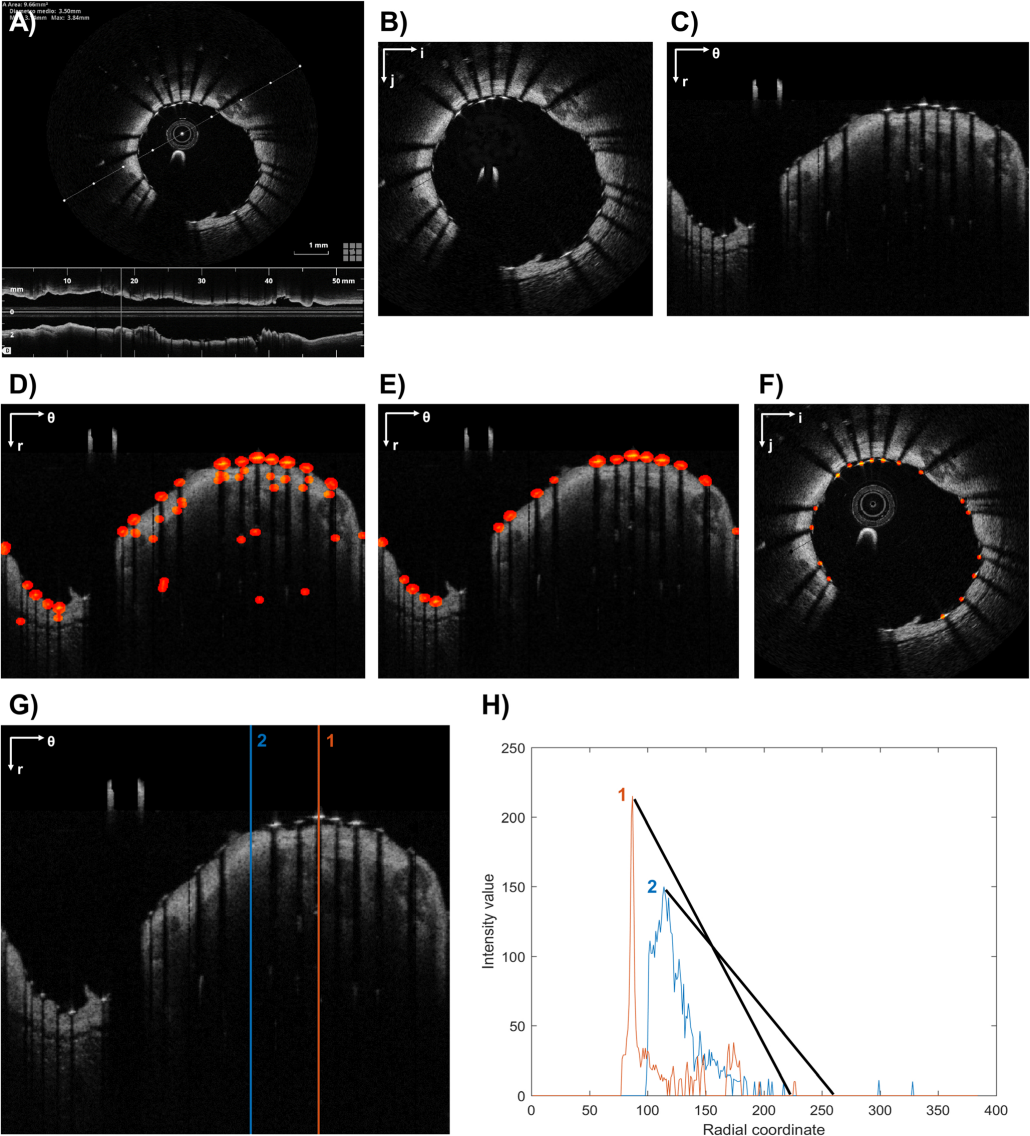
Example of stent strut detection algorithm (**a**–**f**): **a** Original grayscale OCT image. **b** Pre-processed image without OCT catheter. **c** Pre-processed image in polar coordinates. **d** Detected struts (red) after raw detection. **e** Detected struts (red) after removal of false positives. **f** Detected struts (red) after conversion to Cartesian coordinates. Example of strut detection (**g**, **h**): **g** Two A-scans are analyzed. A-scan 1 includes a stent strut while A-scan 2 is only the vessel wall. **h** Corresponding intensity profiles of A-scans 1 and 2. The strut is detected because of the higher slope of the A-scan intensity profile. The polar coordinate system (*r*; *θ*) or the Cartesian coordinate system (*i*; *j*) is indicated on the top left of the images. The example refers to a post-operative OCT frame of a patient treated at the Institute of Cardiology, Catholic University of the Sacred Heart (Rome, Italy), with a Xience Prime stent (Abbott Vascular, USA). The image was processed using the algorithm described in [41, 42]

The workflow for stent strut detection usually comprises three steps: (1) pre-processing, (2) detection of stent strut candidates, and (3) removal of false positives. An example of this workflow is shown in Fig. 3a–f.

As with the lumen segmentation algorithms, pre-processing is necessary to reduce the image noise and prepare the image for the subsequent steps. Comparable procedures to those described in the previous section are usually employed during this step.

Until now, a number of different strategies have been proposed for the detection of stent strut candidates and the removal of false positives. Since metallic struts appear in OCT images as high-reflecting spots (i.e., high-intensity region) followed by a trailing shadow behind them (i.e., low-intensity region) (Fig. 3a), the majority of the algorithms search for these features to detect the stent struts. For instance, Gurmeric et al. [14] and Unal et al. [16] analyzed the angular intensity distribution in Cartesian coordinates to localize the shadows and detect the struts on the shadow rays as the maximum bright intensity pixel group and the most negative deep gradient vectors following such a group. Bruining et al. [21] defined a basic set of features (i.e., mean, maximum, sum of intensity values above the mean) for each A-scan and detected the stent struts by solving a feature-based classification problem with a K-nearest-neighbor method. Lu et al. [23] used a machine learning, classification approach. A classifier was trained with specific features of the images, such as intensity statistics of the strut and trailing shadow regions, to identify candidate pixels of stent struts. Tung et al. [24] first detected the trailing shadows in polar coordinates by computing the cumulative intensity histogram of each A-scan and then applied probability map and morphological operations to segment the stent struts. Ughi et al. [25] identified the A-scans containing a strut candidate based on the presence of high peak intensity, a very fast rise and fall of energy, a significant drop in intensity, and the shadow length. Chiastra et al. [41] and Migliori et al. [42] computed the slope of the line connecting each high-intensity peak (e.g., above the 90th percentile of the intensity histogram of the frame) and the following 30th pixel with low intensity (e.g., below the median of the intensity histogram of the frame), as previously proposed [27]. Since strut pixels usually have a steeper slope than tissue pixels, pixels that are associated with a high slope were classified as strut candidates (Fig. 3g–h). Finally, a probability function that penalizes detection of structures far from the lumen contour was applied to remove the false positives.

While in the previous algorithms the stent strut detection was based only on the intensity profiles of the OCT frames, other studies applied also image filters, such as the gradient or the Laplacian filter. In particular, Kauffmann et al. [17] detected the shadow zones by analyzing the gradient component of the image in polar coordinates and localized the strut position by seeking a rapid decrease in gray intensity beyond the strut. Han et al. [28, 34] applied the Laplacian filter to the image in polar coordinates to extract edges and corners and then identified stent struts by selecting only the pixels with an intensity value greater than a specified threshold. Dubuisson et al. [33] detected the shadows as regions with low intensity close to the lumen contour and the strut positions by combining information of the intensity profile and its gradient with the positions of the shadow zone. Nam et al. [37] built a stent candidate set based on the detection of local maxima along the A-scans and then performed a feature selection process to select the subset of features that generates the best results. During this process, geometrical features (e.g., slope, strut length, mean and variance of the shadow region) and statistical features of the intensity image (e.g., maximum, median, mean intensity) and the second gradient image (e.g., maximum, median, mean amplitude) were taken into account.

Alternative approaches for stent strut detection follow. Xu et al. [20] applied a steerable ridge filter for identifying the struts, which appear like ridge structures in follow-up OCT images with severe in-stent restenosis. Wang et al. [32] proposed an algorithm characterized by the following phases: (1) the probability of stent strut appearance in each A-scan was computed using a Bayesian network; (2) the strut position within the A-scan was reinforced using stent mesh information from adjacent frames; (3) the exact strut depth positions were simultaneously located in the OCT pullback using a graph cut algorithm. O’Brien et al. [38] relied on a method previously developed for OCT images of stented femoral arteries [48], which detects the stent struts by introducing each strut wavelet response into a feature extraction and classification scheme.

**Fig. 4:**
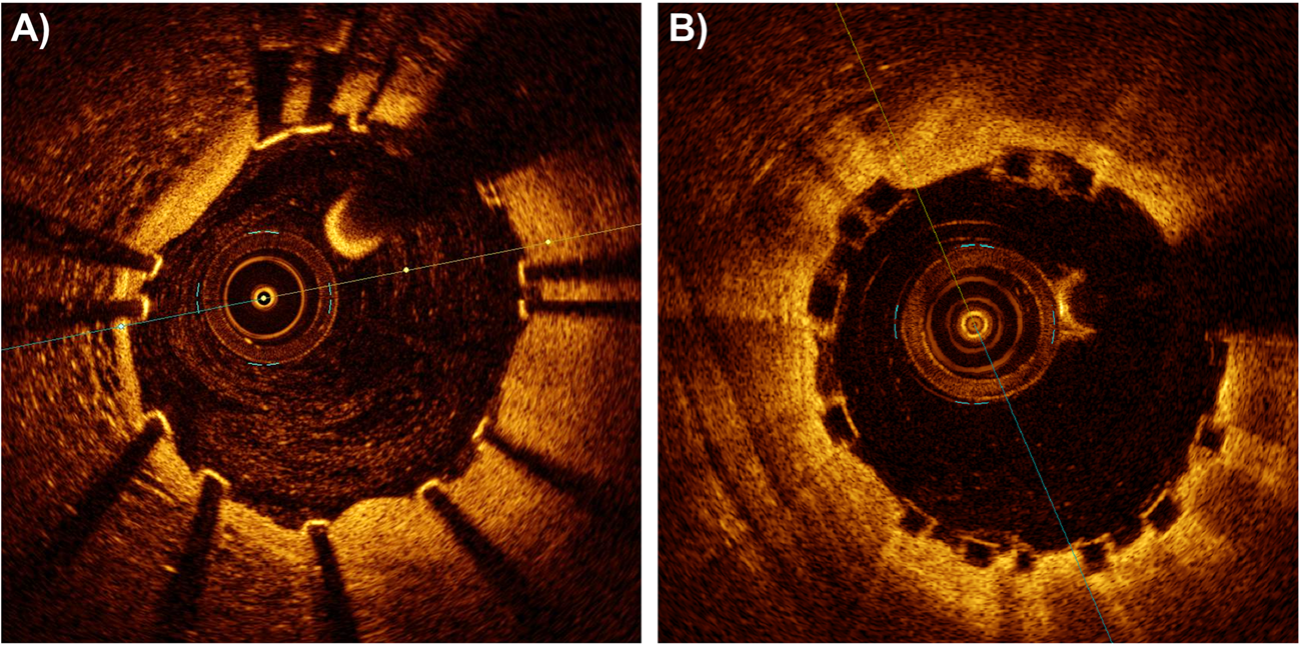
Differences between strut appearance of metallic and polymeric stents in OCT images: **a** post-operative OCT image of a patient treated at the Institute of Cardiology, Catholic University of the Sacred Heart (Rome, Italy), with a Magmaris stent (Biotronik, Germany) (bioresorbable metallic stent). **b** Post-operative OCT image of a patient treated with an Absorb BVS (Abbott Vascular, USA) (bioresorbable polymeric stent). OPTIS™ Stent Optimization Software (St. Jude Medical, USA). Image provided courtesy of St. Jude Medical, Inc.

All previously reported algorithms work with metallic stent struts only and cannot detect polymeric struts, which are characterized by the absence of trailing shadows in the OCT images (Fig. 4) [49]. Currently, only one study has proposed a detection algorithm for polymeric stent struts, specific for the Absorb BVS (Abbott Vascular, USA) [29]. This algorithm detects the black core of the Absorb BVS struts by analyzing the intensity profile and the gradient profile of each A-scan.

### General Observations

The strategies proposed for the detection of lumen contours and stent struts are numerous and various. Many metrics and indexes have been used to assure the reliability of the detection algorithms. However, a direct comparison between the results obtained by the different methods is difficult because a standard validation procedure as well as a common validation dataset were not used [50]. Indeed, the standardization of the validation procedure would enable an easier comparison of the algorithms’ performance between different research groups and the selection of the optimal detection methods.

**Fig. 5:**
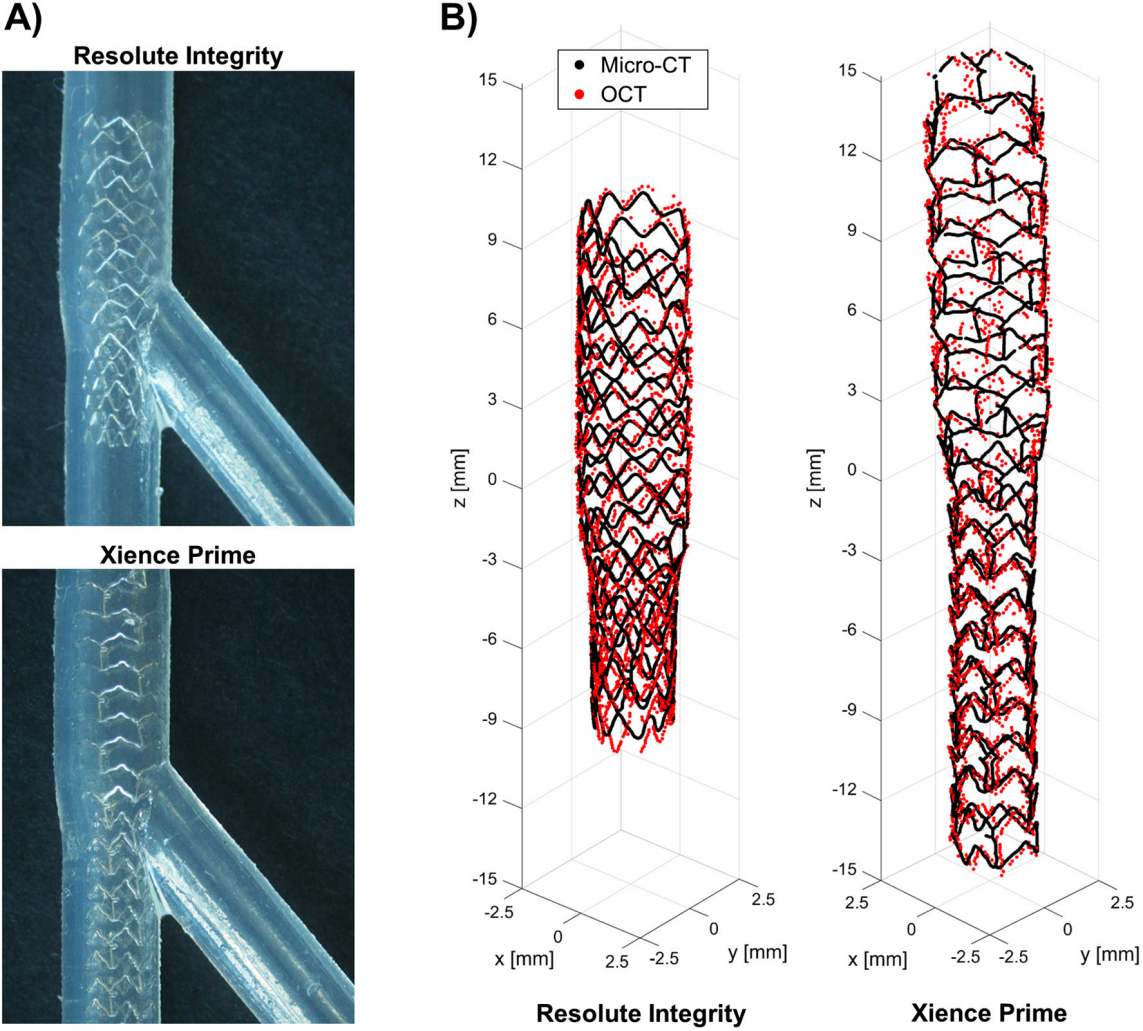
Validation of the stent strut detection algorithm using micro-computed tomography (micro-CT) [41]. **a** Details of Resolute Integrity (Medtronic, USA) (top) and Xience Prime (Abbott Vascular, USA) (bottom) stents deployed in 40° coronary bifurcation phantoms. **b** Superimposition of the stent point clouds obtained using the automatic detection algorithm and micro-CT for the Resolute Integrity (left) and Xience Prime (right) cases. Adapted with permission from [41]

With a few exceptions (Table 1), the detection algorithms were validated against manual segmentation performed by independent expert image readers, which was accepted as ground truth. Different metrics and indexes were analyzed by using standard statistical analysis methodologies. In particular, to evaluate the lumen contour detection algorithms, the similarity indexes (i.e., sensitivity, specificity, Jaccard index, Dice index) and other metrics were calculated, such as the lumen area and the distance between the lumen contours obtained with the automatic and manual segmentations. To assess the reliability of the strut detection algorithms, the similarity indexes, the distance between each automatically segmented strut and the closest manually detected, and the length of apposition (i.e., radial distance between a strut and the lumen border) were computed. Only in one study, the validation of the strut detection algorithm was performed against both manual segmentation and micro-computed tomography (micro-CT) 3D reconstructions [41]. More in detail, a coronary stent was deployed in two silicon bifurcation phantoms by an interventional cardiologist (Fig. 5a). The phantoms were scanned with both OCT and micro-CT. Finally, the stent point clouds obtained by applying the stent strut detection algorithm were compared in 3D against the centerline points of the same stent reconstructed from micro-CT (Fig. 5b).

Until now, all proposed stent strut detection algorithms were developed for metallic stents. As highlighted in Table 1, the only exception is the algorithm by Wang et al. [29] for the detection of the Absorb BVS polymeric struts. Although the implant of this bioresorbable scaffold is currently not recommended because of recent clinical findings of high thrombosis risk [51], the need of detection algorithms for polymeric stents will probably become more important as a result of the extensive development of this technology [52].

The calculation time needed to process OCT images is not a problem for the entire workflow of 3D reconstruction of stented coronary artery models. The calculation time needed to segment each OCT frame ranges between 0.1 s and a few seconds depending on the programming language used for the codes (Table 1). The majority of the algorithms have been developed using Matlab (Mathworks, USA). The conversion of the codes to lower-level languages (e.g., C++) and the use of graphics processing units for the calculation can dramatically reduce the calculation time [34], thus allowing for real-time segmentation of entire OCT pullbacks.

Table 1 also highlights that all the published detection algorithms have been applied to OCT datasets acquired with the LightLab Imaging/St. Jude Medical (USA) OCT imaging system. It is not clear whether those algorithms can process OCT pullbacks obtained with other OCT imaging systems, such as the one of Terumo Corp. (Japan), with the same accuracy.

Finally, it is worth mentioning that most of the commercial OCT systems’ software currently includes semi-automated segmentation algorithms for the detection of lumen contours and stent struts. However, this software has been developed for clinical purposes only. The export of the segmented data (i.e., point clouds of the segmented lumen contours and stent struts), which is necessary for the subsequent 3D reconstruction of the stented vessel, is usually not allowed or restricted to specific clinical centers by the OCT system manufacturer. As opposed to the detection algorithms of the commercial OCT systems, the methods listed in Table 1 are usually more flexible as all the algorithm parameters can be adjusted by the user and the segmentation results can be exported for executing the subsequent steps of the 3D reconstruction process.

## OCT-Based Coronary Artery Models for Fluid Dynamics Analyses

### State of the Art of 3D Geometrical Reconstructions

Since OCT only generates cross-sectional vessel images orthogonal to the OCT catheter, the 3D reconstruction of coronary artery models requires the fusion of OCT data with another imaging technique (i.e., angiography or CT) that allows the extraction of the vessel centerline or the OCT guide-wire pathway (Fig. 1). The different approaches proposed in the literature to reconstruct stented coronary artery models for CFD analyses are described hereinafter.

Two studies [53, 54] combined OCT and CT to create the 3D vessel lumen model. In particular, in Ellwein et al. [53], a preliminary 3D vessel reconstruction from post-operative CT was used to estimate the guide-wire pathway by applying a shortest path algorithm to a graph representation of the artery and assuming that the guide-wire adopts the straightest configuration inside a tortuous artery. Lumen contours extracted from post-operative OCT were then positioned orthogonal to the guide-wire pathway and smoothly connected to obtain the final lumen model. The stent was drawn inside the artery by following a methodology that creates an idealized model of a thick stent matching the vessel geometry and generates the fluid domain by subtracting the stent from the vessel lumen solid model. Chiastra et al. [54] adopted the previously described method to reconstruct the pre-operative vessel lumen model and then performed finite element analyses of stent deployment to obtain the post-operative stented geometry.

In several works [55–63], the stented vessel geometry was generated directly from OCT without the need of virtual stent implantation. The following method was used: (1) lumen contours accounting for the stent strut presence were manually delineated in each OCT frame; (2) the centroid of each stented lumen contour was calculated and used to place the OCT frames orthogonal to vessel centerline extracted from two angiographic projections; the side branches were chosen as landmarks for estimation of the relative axial twist of OCT frames to properly orient each contour; (3) the contours were connected by creating a non-uniform rational B-spline surface representing the stented artery. Although the resultant stent geometries were corrugated and hardly continuous, this procedure allowed the reproduction of the real, overall stent shape within the artery, unlike the previous two studies [53, 54].

**Fig. 6:**
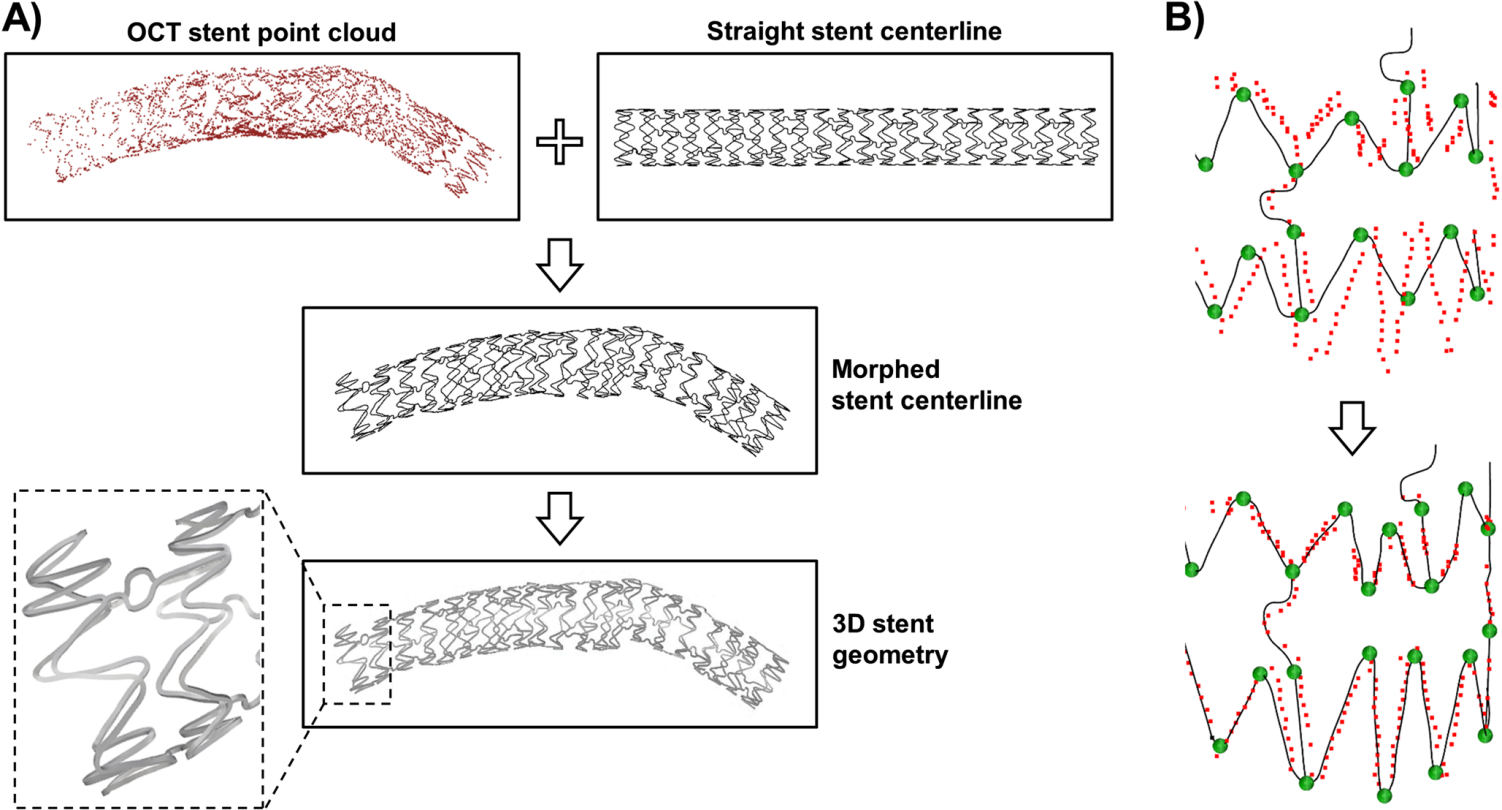
Example of morphing procedure for the creation of a patient-specific stent model from OCT data. **a** The stent skeleton in straight expanded configuration (i.e., the straight stent centerline) is morphed on the OCT stent point cloud to generate the deployed configuration of the stent centerline (i.e., morphed stent centerline) and subsequently the 3D stent geometry. **b** Morphing of the centerline by using handles to minimize its distance with the OCT stent point cloud. The example refers to a post-operative OCT dataset of a patient treated at the Institute of Cardiology, Catholic University of the Sacred Heart (Rome, Italy), with a Xience Prime stent (Abbott Vascular, USA). The morphing procedure is based on the method described in [42]

Further works proposed advanced methods for patient-specific 3D stent reconstruction. Gogas and colleagues [64, 65] developed a method for the reconstruction of the Absorb BVS based on an interactive scaffold pattern interpretation algorithm and the fusion of OCT and angiographic data. More in detail, an operator classified the strut points detected from OCT as belonging to the rings and the links of the bioresorbable scaffold using an in-house Matlab application for pattern interpretation. Subsequently, the strut points of each ring/link were positioned along the vessel centerline extracted from angiography and interpolated using cubic splines in order to obtain a continuous 3D scaffold skeleton. Finally, a series of rectangles representing the scaffold cross sections were automatically placed along each skeleton line and connected to create the final scaffold model. Differently, O’Brien et al. [38] and Migliori et al. [42] used prior stent design knowledge to create the patient-specific stent model. For instance, in Migliori et al. [42], the deployed stent geometry was reconstructed starting from the OCT strut point cloud using the following morphing procedure (Fig. 6): (1) the stent skeleton in its straight free-expanded configuration was created; (2) the skeleton lines were morphed on points that belong to the stent point cloud so that their distance was minimized; (3) the morphed skeleton was used to generate the 3D patient-specific stent geometry by connecting cross-section curves placed along the skeleton lines.

### State of the Art of CFD Simulations

Table 2 summarizes the recent studies on fluid dynamics of stented coronary artery models reconstructed from OCT, including information on the simulation settings (i.e., blood description and boundary conditions) and the main findings. While some of them are proof-of-concept studies that demonstrate the feasibility of using these models to perform CFD simulations [64, 65] or present a novel modeling strategy in detail [38, 42, 53, 66], others [55–57] investigated the relationships between WSS and neointimal hyperplasia in patients treated with Absorb BVS. Although the number of investigated patients was small, an inverse correlation between the logarithmic transformed WSS and the neointimal thickness at 6-months follow-up was found, showing that low WSS seems to be one of the factors promoting in-stent restenosis in patients treated with Absorb BVS. Serruys’ group also investigated the local hemodynamic microenvironment in porcine coronary segments with Absorb BVS [58], the hemodynamic changes between baseline and a 5-year follow-up in a patient treated with the Absorb BVS [62], and the impact of underexpanded and overexpanded bioresorbable scaffolds on hemodynamics in the long term [63]. Furthermore, they used CFD simulations as a preclinical assessment tool by comparing different bioresorbable scaffolds implanted in porcine coronary arteries, such as the Absorb BVS, the AterioSorb (Arterious, UK), and the Mirage BMRS (Manli Cardiology Ltd., Singapore), from the fluid dynamics viewpoint [59–61]. Finally, Chiastra et al. [48] developed an OCT-based sequential modeling strategy (i.e., structural simulations of stent deployment followed by CFD analyses) that can be applied as a virtual bench testing tool to compare different stent designs, deployment locations, and stenting techniques in patient-specific anatomies. In particular, the biomechanical effects (e.g., malapposition rate and stented lumen areas exposed to low WSS) of different stent designs and stent deployment locations were analyzed in two patient-specific coronary bifurcations.

**Table 2:** List of published studies on fluid dynamics simulations of stented coronary artery models reconstructed from OCT

First author, year [reference]	Number of cases (stent type)	Follow-up	3D model reconstruction	Software (analysis type)	Blood description	Boundary conditions	Notes
Ellwein et al., 2011 [53]	1 patient (DES)	Month 6	CT + OCT	ALTAIR LesLib (pulsatile)	Newtonian fluid (*μ* = 0.004 Pa·s), *ρ* = 1060 kg/m^3^	Inlet: Womersley velocity profile (*Q*_in_ from cardiac output) Outlets: three-element Windkessel representation	Method for patient-specific coronary artery reconstruction for CFD analyses
Gogas et al., 2013 [64]	1 patient (Absorb BVS)	No	2 angio + OCT	LifeV (ND)	ND	ND	Proof-of-concept study
Papafaklis et al., 2013 [55]	1 patient (Absorb BVS)	Month 6	2 angio + OCT	ANSYS CFX (steady state)	Newtonian fluid (*μ* = 0.0035 Pa·s), *ρ* = 1050 kg/m^3^	Inlet: flat velocity profile (FCM) Outlet: 0 Pa	Higher relationship between WSS and NIH for OCT-based than IVUS-based analysis
Bourantas et al., 2014 [57]	12 patients (Absorb BVS)	Month 12	OCT	ANSYS CFX (steady state)	Newtonian fluid (*μ* = 0.0035 Pa·s), *ρ* = 1050 kg/m^3^	Inlet: flat velocity profile (FCM) Outlet: 0 Pa	Inverse correlation between WSS and NIH (*r* = 0.451, *p* < 0.001)
Bourantas et al., 2014 [56]	6 patients (Absorb BVS)	Month 6 or 12	2 angio + OCT	ANSYS CFX (steady state)	Newtonian fluid (*μ* = 0.0035 Pa·s), *ρ* = 1050 kg/m^3^	Inlet: flat velocity profile (FCM) Outlet: 0 Pa	Fusion of OCT with angiography better than IVUS-based reconstructions. Inverse correlation between WSS and NIH (*r* = −0.52 ± 0.19, *p* = 0.028)
Gogas et al., 2016 [65]	1 patient (Absorb BVS)	No	2 angio + OCT	LifeV (ND)	ND	ND	Proof-of-concept study
Chiastra et al., 2016 [54]	2 patients (DES)	No	CT + OCT + finite element analysis of stent deployment	SimVascular (pulsatile)	Newtonian fluid (*μ* = 0.004 Pa·s), *ρ* = 1060 kg/m^3^	Inlet: Womersley velocity profile (*Q*_in_ from cardiac output) Outlets: three-element Windkessel representation	Workflow for pre-interventional planning
O’Brien et al., 2016 [38]	1 pig (BMS)	No	2 angio + OCT + stent morphing	ANSYS Fluent (steady state)	Non-Newtonian fluid (Carreau model), *ρ* = 1060 kg/m^3^	Inlet: parabolic velocity profile (*v*_mean_ = 0.25 m/s) Outlet: 0 Pa	Enhanced method to characterize implant microenvironments (stent geometry and fluid dynamics)
Tenekecioglu et al., 2016 [60]	2 pigs (Absorb BVS, Mirage BMRS)	No	2 angio + OCT	ND (steady state and pulsatile)	ND	Inlet: FCM Outlet: 0 Pa	Absorb BVS is associated to lower WSS than Mirage BMRS
Huang et al., 2017 [66]	1 patient (Absorb BVS)	No	2 angio + OCT	ANSYS (pulsatile)	Newtonian fluid (*μ* = 0.0035 Pa·s), *ρ* = 1060 kg/m^3^	Inlet: Womersley velocity profile (*Q*_mean_ = 250 ml/min) Outlet: 0 Pa	Hemodynamic characterization of the Absorb BVS in a patient-specific environment
Tenekecioglu et al., 2017 [58]	6 pigs (Absorb BVS)	Month 1	2 angio + OCT	ANSYS Fluent (steady state)	Newtonian fluid (*μ* = 0.0035 Pa·s), *ρ* = 1050 kg/m^3^	Inlet: flat velocity profile (FCM) Outlet: 0 Pa	Absorb BVS induces fluctuations of WSS, which are related to pathologic findings in stented segments
Tenekecioglu et al., 2017 [61]	1 pig (ArterioSorb-95 μm, ArterioSorb-120 μm)	No	2 angio + OCT	ND (steady state)	ND	ND	Lumen exposure to low WSS is lower for the ArterioSorb-95 μm than the ArterioSorb-120 μm
Tenekecioglu et al., 2017 [59]	2 pigs (Absorb BVS, ArterioSorb-95 μm)	No	2 angio + OCT	ND (pulsatile)	ND	ND	ArterioSorb resulted in higher WSS than Absorb BVS during all phases of coronary flow
Thondapu et al., 2017 [62]	1 patient (Absorb BVS)	Month 60	2 angio + OCT	ND (pulsatile)	Non-Newtonian fluid (model ND), ρ ND	ND	Lumen exposure to low WSS decreases after 5 years post-implantation
Torii et al., 2017 [63]	2 patients (Absorb BVS)	Month 60	2 angio + OCT	ANSYS Fluent (steady state)	Newtonian fluid (*μ* = 0.0035 Pa·s), *ρ* = 1050 kg/m^3^	Inlet: flat velocity profile (FCM) Outlet: 0 Pa	WSS is different in the case of underexpanded or overexpanded stent
Migliori et al., 2017 [42]	1 coronary artery phantom (BMS)	No	2 angio + OCT + stent morphing	ANSYS Fluent (pulsatile)	Non-Newtonian fluid (Carreau model), *ρ* = 1060 kg/m^3^	Inlet: flat velocity profile (*Q*_mean_ = 45.15 ml/min) Outlet: 0 Pa	Validated method to reconstruct high-fidelity stented geometries for CFD analyses

*BMS* bare metal stent, *DES* drug-eluting stent, *Absorb BVS* Absorb bioresorbable vascular scaffold (Abbott Vascular), *Mirage BMRS* Mirage bioresorbable micro-fiber scaffold (Manli Cardiology Ltd., Singapore), *ArterioSorb* bioresorbable scaffold (Arterius), *angio* angiography, *FCM* frame count method, *Q*_in_ inlet flow rate, *NIH* neointimal hyperplasia, *IVUS* intravascular ultrasound, *ND* not declared

### General Observations

Among the different strategies proposed for the 3D reconstruction of stented coronary artery models, that based on prior stent design knowledge [38, 42] seems to be the most promising. This procedure allows the creation of stent geometries with higher accuracy as compared to the 3D reconstruction method applied in [55–63]. In particular, the strategy based on prior stent design knowledge is able to generate continuous stent geometries and to reproduce the exact stent strut cross-sectional shape. These stent geometry characteristics, which cannot be obtained with other 3D reconstruction methods [55–63], are crucial for getting reliable hemodynamic results in the microenvironment of the stent struts. Indeed, the WSS calculation is highly dependent on the local geometry as the WSS is defined as the tangential force per unit area generated by blood flow along the vessel lumen. Small geometrical differences at the stent strut level can lead to important differences on the local WSS distribution. Furthermore, this stent reconstruction method is not limited to the Absorb BVS as in [64, 65] but can be applied to all types of coronary stents regardless of the stent design and strut cross-sectional shape (i.e., rectangular or circular).

Only Migliori et al. [42] validated their reconstruction method. In their work, a 3D-printed coronary artery phantom with a deployed stent was imaged with both OCT and micro-CT. The stented geometry reconstructed from OCT was compared against that obtained from micro-CT, chosen as ground truth. An agreement between the two geometries was found. The results highlighted the importance of using side branches as landmarks for the correct orientation of the stent struts and lumen point clouds along the vessel centerline. The more the landmarks, the less the twist angle error between stent and lumen contours. Additionally, the side branches can be used to define the correct axial position of the contours along the vessel centerline. The recent introduction into the market of OCT imaging systems able to co-register the OCT pullback to angiography [50] will be decisive to improve the contours’ axial positioning.

To exclude the influence of the heart motion on the validation process, Migliori et al. [42] fabricated a rigid coronary artery phantom. However, heart motion artifacts can affect in vivo OCT pullbacks, resulting in 3D reconstructions with vessel segments longer or shorter than the real ones (i.e., elongated or compressed segments). This issue has not been faced so far.

As regards the CFD simulations, the definition of patient-specific boundary conditions is a critical aspect. In vivo measurement of pressure and velocity at different locations of the coronary tree is feasible by using dual-sensor pressure and Doppler velocity wires. Nevertheless, this procedure is barely performed in the clinical routine because it is highly invasive. Alternatively, the inlet boundary condition can be defined by means of the frame count method [55–58, 60]. This technique consists of estimating the inlet flow rate by counting in the angiographic projections the number of frames required for the contrast agent to pass from the inlet to the outlet of a coronary segment free of side branches. The main limitation of the method is the low frequency of the angiographic acquisitions (e.g., 15 frame/s), which results in a low number of frames usable for the flow-rate estimation. In the absence of in vivo measurements, the following strategies can be adopted to define the outlet boundary conditions [67]: (1) traction-free (i.e., zero pressure) boundary condition when side branches are not present; (2) scaling law, which allows computing the flow split between the different branches; (3) coupling of the 3D domain with a lumped-parameter model (i.e., 0D model) representing the downstream vasculature. By applying the zero pressure outlet boundary condition, the WSS results along the lumen can substantially deviate from the true values because the flow division between the vessel branches and the resultant velocity field depend only on the intrinsic resistances from the main vessel geometry and its branches. Conversely, the other two strategies can lead to a more correct estimation of WSS patterns as a more realistic flow division is prescribed at the outlets of the coronary artery model. Several scaling laws (e.g., Murray’s law, HK model, etc.) have been proposed until now [68]. While Murray’s law (${Q}_{D_2}/{Q}_{D_1}={\left({D}_2/{D}_1\right)}^3$, where *Q*_1_ and *Q*_2_ are the flow rates and *D*_1_ and *D*_2_ the diameters of the bifurcation branches) is based on the principle of minimum energy, the HK model [69] (${Q}_{D_2}/{Q}_{D_1}={\left({D}_2/{D}_1\right)}^{7/3}$) is based on the assumption of a fractal-like branching pattern in a tree structure. Another common scaling law is that proposed by van der Giessen et al. [70] (${Q}_{D_2}/{Q}_{D_1}={\left({D}_2/{D}_1\right)}^{2.27}$), which was derived from the fitting of blood flow measurements in human coronary arteries. A recent study demonstrated in ten coronary bifurcations that the normalized WSS pattern was well captured when van der Giessen’s scaling law was applied as compared to an outflow boundary condition based on myocardial flow measurement derived from computed tomography perfusion imaging [71]. The use of 3D-0D coupled models allows investigating the entire circulation (closed-loop 3D-0D model), including the heart and coronary circulation (e.g. [72, 73]), or the coronary artery segment of interest with its downstream vasculature (open-loop 3D-0D model) (e.g. [54, 74, 75]). The recent software advancements and continued increase of computation power have made possible a wider use of these models. However, the estimation of the lumped parameters (i.e., resistances, compliances, and inductances) remains the main issue when in vivo measurements are not available [76].

In all the CFD simulations of Table 2, the lumen and stent surfaces were considered as rigid walls and the coronary artery motion caused by the heart contraction was neglected. Chiastra et al. [77] demonstrated that the rigid-wall assumption is acceptable for the stented segment by comparing an idealized fluid-structure interaction stented model against the corresponding rigid-wall one. Conversely, the impact of the heart contraction on the hemodynamics is still under debate [67] and it is unclear whether a fixed model is reliable enough for the calculation of time-averaged WSS.

The time required for 3D reconstruction and subsequent CFD simulation is an important issue, which currently limit the use of OCT-based fluid dynamics models in large population studies. In particular, the 3D reconstruction methods are highly time demanding. For instance, the method by Migliori et al. [34] requires more than one day for each reconstruction. This time might be dramatically reduced by automating the stent morphing procedure, which is the most time-consuming step. The simulation time is a less critical point because CFD simulations can be efficiently performed on supercomputers. Indeed, commercial software such as Fluent or CFX (ANSYS Inc., USA) are highly scalable and allow transient analyses in few hours on hundred computing cores.

## Conclusions and Future Perspectives

This review analyzed the workflow for the creation of CFD models of stented coronary arteries starting from patient-specific OCT images. The available algorithms for lumen contour and stent strut detection from OCT as well as the 3D reconstruction methods of stented geometries were discussed. Furthermore, the current CFD studies that investigate the local hemodynamics of OCT-based stented coronary artery geometries were reported.

The current methodologies have been applied to few animal or human coronary artery cases as tools for better understating in-stent restenosis and its relationship with abnormal hemodynamics, and comparing different stent designs, stent positions, or stenting techniques from the fluid dynamic viewpoint. The application of these methodologies to large population studies is possible. However, efforts of the scientific community are still requested to improve some steps of the 3D reconstruction, in particular the stent reconstruction, which is mainly manual and time-consuming. Moreover, the definition of standards for the validation of the detection algorithms and the reconstruction methods will be crucial for a widespread clinical use of the OCT-based models for possible future in silico clinical trials.

### Sources of Funding

Susanna Migliori is supported by the European Commission through the H2020 Marie Sklodowska-Curie European Training Network H2020-MSCA-ITN-2014 VPH-CaSE, www.vph-case.eu, GA No. 642612.

## Abbreviations

FDA: Food and Drug Administration
OCT: Optical coherence tomography
3D: Three-dimensional
CFD: Computational fluid dynamics
WSS: Wall shear stress
CT: Computed tomography
Micro-CT: Micro-computed tomography
